# Expression of a Vacuole-Localized BURP-Domain Protein from Soybean (SALI3-2) Enhances Tolerance to Cadmium and Copper Stresses

**DOI:** 10.1371/journal.pone.0098830

**Published:** 2014-06-05

**Authors:** Yulin Tang, Yan Cao, Jianbin Qiu, Zhan Gao, Zhonghua Ou, Yajing Wang, Yizhi Zheng

**Affiliations:** 1 Shenzhen Key Laboratory of Microbial and Gene Engineering, College of Life Sciences, Shenzhen University, Shenzhen, Guangdong, People’s Republic of China; 2 The Key Laboratory for Marine Bioresource and Eco-environmental Science, College of Life Sciences, Shenzhen University, Shenzhen, Guangdong, People’s Republic of China; Iwate University, Japan

## Abstract

The plant-specific BURP family proteins play diverse roles in plant development and stress responses, but the function mechanism of these proteins is still poorly understood. Proteins in this family are characterized by a highly conserved BURP domain with four conserved Cys-His repeats and two other Cys, indicating that these proteins potentially interacts with metal ions. In this paper, an immobilized metal affinity chromatography (IMAC) assay showed that the soybean BURP protein SALI3-2 could bind soft transition metal ions (Cd^2+^, Co^2+^, Ni^2+^, Zn^2+^ and Cu^2+^) but not hard metal ions (Ca^2+^ and Mg^2+^) *in vitro*. A subcellular localization analysis by confocal laser scanning microscopy revealed that the SALI3-2-GFP fusion protein was localized to the vacuoles. Physiological indexes assay showed that *Sali*3-2-transgenic *Arabidopsis thaliana* seedlings were more tolerant to Cu^2+^ or Cd^2+^ stresses than the wild type. An inductively coupled plasma optical emission spectrometry (ICP-OES) analysis illustrated that, compared to the wild type seedlings the *Sali*3-2-transgenic seedlings accumulated more cadmium or copper in the roots but less in the upper ground tissues when the seedlings were exposed to excessive CuCl_2_ or CdCl_2_ stress. Therefore, our findings suggest that the SALI3-2 protein may confer cadmium (Cd^2+^) and copper (Cu^2+^) tolerance to plants by helping plants to sequester Cd^2+^ or Cu^2+^ in the root and reduce the amount of heavy metals transported to the shoots.

## Introduction

The BURP domain was defined by Hattori *et al.* following those proteins in which the conserved domain was first identified: the BNM2, USPs, RD22 and PG1beta [1]. The BURP proteins are characterized by the BURP domain in the C terminus and can be classified into four subfamilies: B, U, R and P [2], [3]. These proteins are plant kingdom specific-proteins. A number of BURP genes have been identified. In addition to those genes that were sporadically isolated in special tissues, developmental stages or conditions [4], [5], [6], [7], [8], 5 members in *Arabidopsis thaliana* [9], 17 in *Oryza sativa* L. [10], 23 in *Glycine max* [11], 15 in *Zea mays*, 11 in *Sorghum vulgare* [12] and 18 in *Populus trichocarpa* [13] were identified genome-widely. The expression of these genes exhibits different temporal and spatial profiles [7], [10], and some of them can be regulated by various stress treatments [10], [11], [14], suggesting that BURP genes might play diverse roles in plant development and stress responses.

A few studies have explored the function of BURP proteins. Some BURP proteins function in particular intracellular compartments. For example, AtUSP in *Arabidopsis thaliana* [9] and BNM2 in *Brassica napus* [15], are most likely localized in cellular compartments such as the Golgi cisternae, dense vesicles, prevaculoar vesicles and protein storage vacuoles. These proteins are related to seed protein synthesis and storage and seed development. Some other BURP proteins are likely localized in the cel1 wall or apoplasts and interact with cell wall components. For instance, OsBURP16 in rice is localized in the cell wall; its overexpression causes pectin degradation and affects cell wall integrity, resulting in the decreased tolerance to abiotic stresses [16]. The cotton AtRD22-Like 1 protein GhRDL1 interacts with an α- expansin GhEXPA1 in the cel1 wall and functions in cell wall extension [17]. The soybean GmRD22 interacts with a cell wall peroxidase in apoplasts. This protein affects cell wall integrity and alleviates salinity and osmotic stress in plants [18]. Thus, different BURP proteins present diverse subcellular localizations and play different functional roles. Nevertheless, the molecular functions of most BURP proteins, especially that of their BURP domain, remain unclear.

BURP domain proteins are characterized by their C-terminal BURP domain which possesses conserved features, including four repeated cysteine-histidine motifs that were arranged as: CH-X_10_-CH-X_25–27_-CH-X_23–24_-CH (X can be any amino acid) [1], [3] and additional two cysteine residues. In view of these characteristics, we assume that, similar to Cys-rich proteins [19], these proteins might interact with some metal ions with high affinity due to their cysteine residues. SALI3-2 is a member in the U subfamily of the BURP family in soybean. Our previous studies demonstrated that Cu^2+^ could bind to SALI3-2 and alter the conformation of SALI3-2. In addition, the *Sali*3-2-transgenic Arabidopsis seedlings were more tolerant to excessive Cu^2+^ stress [20], indicating that the SALI3-2 is associated with heavy metal tolerance in plants. However, new questions should be asked and are as following: can SALI3-2 bind other soft transition metal ions besides Cu^2+^? Can the expression of *Sali*3-2 in transgenic plants confer to them tolerance to other heavy metal ions? In this study, we confirmed the *in vitro* interaction of SALI3-2 with either Cu^2+^ and some other soft metal ions (Cd^2+^, Co^2+^, Ni^2+^ and Zn^2+^) using immobilized metal ion affinity chromatography (IMAC). We further analyzed the subcellular localization of SALI3-2 and the tolerance of *Sali*3-2-transgenic Arabidopsis to the stresses of the typical soft metal ions (Cd^2+^ and Cu^2+^). The mechanisms of SALI3-2 functioning in heavy metal tolerance are discussed.

## Materials and Methods

### Construction of pET28a/*Sali*3-2c for SALI3-2 Expression in *E. coli*

The region encoding the SALI3-2 fragment without a signal peptid (named SALI3-2C) was amplified by polymerase chain reaction (PCR) with the template pET28a-*Sali*3-2 carrying the full-length *Sali*3-2 gene and oligonucleotide primers containing the NdeI and a XhoI sites: sense primer 5′- gctcttcatatggagagccatgtccatgc -3′ and antisense primer 5′- tgcgctcgagttaaacaacaacgttagtctgatag -3′ (restriction sites underlined). The resulting PCR product as well as the plasmid pET28a were digested with NdeI and XhoI and further ligated. The constructed vector pET28a-*Sali*3-2c was transformed into *E. coli* cells (strain BL21 Star) for protein expression. The encoded protein SALI3-2C was fused with a 6×His tag at its N-terminus (6×His-SALI3-2C) and can be cleaved off by the protease thrombin.

### Expression and Purification of the SALI3-2C Protein

*E. coli* cells transformed with pET28a-*Sali*3-2c were cultured at 37°C and 180 rpm until the OD_600_ reached 0.6∼0.8. The 6×His-SALI3-2C protein was then induced by the addition of 0.5 mM isopropyl-β-Dthiogalactopyranoside (IPTG) into the cultures. *E. coli* cells were cultured for additional 4 h and then collected by centrifugation at 10,000 rpm for 15 min. The harvested cells were then re-suspended and disrupted in Tris–HCl buffer (50 mM Tris–HCl, 1 mM EDTA and 100 mM NaCl, pH 8.0) by 30 min of sonication, further centrifuged at 10,000 rpm for 15 min. The pellet was re-suspended again in 5 mL of Tris–Urea buffer (0.1 mM Tris–HCl, 6 mM urea, 1 mM PMSF and 1 mM EDTA, pH 8.0) and dissolved at room temperature for at least 1 h. The resulting suspension was centrifuged at 10,000 rpm for 15 min. The supernatant was harvested and passed through a Ni-sephorose column (AKTA, Amersham Biosciences, Tokyo, Japan) and washed with a buffer containing decreasing concentrations of urea (20 mM Tris–HCl, 1 mM EDTA and 6∼2 mM urea, pH 8.0) for protein renaturation. Finally, the column was washed with washing buffer (200 mM NaCl, 50 mM Tris–HCl and 100 mM imidazole, pH 8.0) and the 6×His-SALI3-2C protein was eluted with buffer containing 500 mM imidazole. The eluted 6×His-SALI3-2C solution was passed through a desalinization column (AKTA, Amersham Biosciences, Tokyo, Japan) to exclude salt and imidazole.

SALI3-2C protein was derived from the cleavage of the 6×His-SALI3-2C protein by protease thrombin (Sigma). Thrombin was mixed with the 6×His-SALI3-2C protein at a ratio of 3 U/mg and incubated at 4°C for overnight. Thereafter, the mixture was passed through a Ni-sepharose column. Thrombin did not bind to the Ni-sepharose and flowed through the column with the balance buffer; SALI3-2C was then recovered by passing through a balance buffer with 60 mM imidazole, and the cleaved 6×His tag was bound tightly to the Ni-sepharose which would be eluted by 500 mM imidazole. The protein concentration was determined by the absorbance at 280 nm.

### Metal-chelating Affinity Chromatography

The interactions between SALI3-2 and the metal ions were analyzed by IMAC using HiTrap Chelating HP (5 mL, Amersham Pharmacia Biotech, Tokyo, Japan) according to Hara *et al.* [21]. The columns were charged by applying 5 mL 100 mM MgCl_2_, CaCl_2_, CdCl_2_, CoCl_2_, NiCl_2_, ZnCl_2_ or CuCl_2_. After washing out the excess metal with deionized water, the column was equilibrated with 20 µM Tris–HCl buffer at pH 8.0 containing 500 mM NaCl (equilibrium buffer). A HiTrap Chelating HP column without metal charging was used as a control. A 5 mL sample of SALI3-2C at approximately 2 µM was applied to the column charged with or without metals. The unbound protein was run and washed out with the equilibrium buffer. The bound protein was eluted by the addition of 5 mL 250 mM EDTA. A total of 12 µL of each fraction was subjected to SDS–PAGE analysis.

### Subcellular Localization Detection of SALI3-2 in Tobacco Cells

To investigate SALI3-2 localization, the *Sali3*-*2* gene was inserted into the pCAMBIA1302 vector at the NcoI and SpeI sites to construct the fusion gene *Sali*3-2-m*Gfp* under the control of the CaMV 35S promoter. This construct and pCAMBIA1302 (as control) were introduced into tobacco cells BY2 (kindly provided by Prof. Liwen Jiang of the Department of Biology and Molecular Biotechnology Program, Chinese University of Hong Kong) mediated by *Agrobacterium* LBA 4404 [22]. Using the fluorescent dye FM4-64 as the membrane marker, the expression of SALI3-2-mGFP and mGFP was analyzed by confocal laser scanning microscopy using Olympus FV1000 (Olympus, Tokyo, Japan). The filter sets that were used for mGFP were excitation 488 nm and emission 510 nm and for FM4-64 were excitation 543 nm and emission 572 nm. To visualize the vacuoles, the cells were observed under bright field after a 5 min immersion in 33 µg/mL neutral red solution.

### Stable Transformation of *Arabidopsis thaliana* (Ecotype Columbia) Using a 35S-*Sali*3-2 Cassette

A cauliflower mosaic virus (CaMV) *35S*-*Sali*3-2 cassette was cloned into the pCAMBIA-1300 vector and transformed into *Arabidopsis thaliana* ecotype Columbia, *via* floral dip [23] mediated by *Agrobacterium tumefaciens* (LBA 4404). The T0 seeds were germinated on 1/2 MS (Murashige–Skoog) medium-containing agar plates under hygromycin selection to obtain T1 resistance plants. The homozygous transgenic lines were further selected according to a segregation ratio of 3∶1 of hygromycin resistance of the T2 generation and homozygous in the T3 generation. The expression of *Sali*3-2 in homozygous transgenic lines was further confirmed by RT-PCR using total RNA extracted from the young leaves of 3-week-old plants. The primers used in these reactions for *Sali*3-2 were 5′-cacacaagcttcaatggaatttcgatgctca-3′ and 5′-tgcgctcgagttaaacaacaacgttagtctgatag-3′ and for tubulin were 5′-ccgatgttgctgtcctcttgg-3′ and 5′-catcaccacggtacatcag-3′.

### Seedling Growth Analysis of *Arabidopsis thaliana* after Heavy Metal Exposure

The seeds of the T3 homozygous transgenic lines and wild-type plants were germinated on 1/2 MS medium-containing agar plates (control), or plates that were supplemented with 50 µM or 75 µM CdCl_2_ or 75 µM or 100 µM CuCl_2_. The plates were incubated at 21°C under a light intensity of 50 µE m^–2 ^s^–1^ at 8 h light/16 h dark. After cultivation for 3 d, the seeds germination ratio was calculated, and after 7 d, the phenotypes of the seedlings, especially the root growth, were recorded.

### Measurement of Metal Contents

Two-week-old *Arabidopsis thaliana* seedlings grown on 1/2 MS agar plates were transferred into 1/2 MS solution medium containing 75 µM CdCl_2_ or 100 µM CuCl_2_ and grown for an additional 2 days. The shoots and roots of the plants were collected, carefully washed with deionized water and dried. The methods for measuring the contents of copper and cadmium followed those mentioned in Narukawa et al.’s paper [24]. Briefly, the dried samples were digested with 15.3 M HNO_3_ in a microwave digestion system ETHOS One (Milestone Inc., Milan, Italy). The digested samples were then diluted with 0.5 N HNO3 and analyzed using an Inductively Coupled Plasma Optical Emission Spectrometer (ICP-OES, OPTIMA 7000DV, Perkin Elmer, NY, USA).

### Transcript Measurement

To determine the transcriptional levels, quantitative RT-PCR (qRT-PCR) was performed. The water-soaked seeds of *Glycine max L.* (*var.* Bainong 6#, kindly provided by the Institute of Agriculture Science in Baicheng City, Jilin Province, P. R. China) were germinated on wet filter paper for 4 days, after which the seedlings were transferred into 1/2 Hoagland’s nutrient solution for an additional 6 days of growth. The roots, stem and leaves were collected separately for total RNA isolation using TRIzol Reagent (Invitrogen, Carlsbad, CA, USA). cDNA was synthesized from 2 µg RNA using PrimeScript RT reagents Kit (TaKaRa, Dalian, China). qRT-PCR was performed on Real-Time PCR Systems ABI7300 (ABI) with SYBR Premix Ex Taq (TaKaRa, Shiga, Japan) according to the manufacturer’s instructions. The gene expression levels were normalized to those of *Tubulin.* The *p*rimers of *Tubulin* and *Sali3*-*2* were the same as those used in RT-PCR described above. Three biological replications were repeated.

### Statistical Analysis

The statistical tests were performed using the data analysis program SPSS. The significant differences between the lines were analyzed using a one-way ANOVA Tukey’s HSD test.

## Results

### SALI3-2 is able to Bind Several Types of Soft Metal Ions

**Figure 1:**
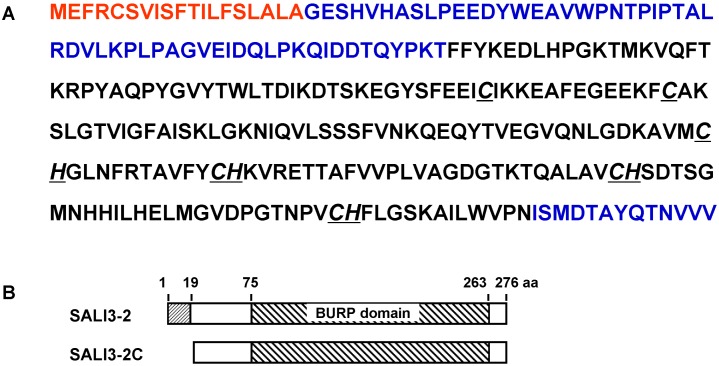
Sequences and structure of SALI3-2 [Glycine max]. A. Sequences of SALI3-2. The accession number is AAB66369. The regions in red, blue and black letters are the putative signal peptide, variable region and BURP domain, respectively. B. Schematic diagram of the structure of SALI3-2 and SALI3-2C.

A search of the sequence databases with SALI3-2 revealed that SALI3-2 contains 276 amino acids and exhibits a modular structure of a BURP protein consisting of a putative signal peptide of 19 amino acids [25], a variable region of 56 amino acids, and a BURP domain of 188 amino acids followed by a C-terminal tail of 13 amino acids (Figure 1).

For those proteins possessing a signal peptide, the signal peptide is usually cleaved off during protein biogenesis to produce the mature protein. Therefore, to obtain the SALI3-2 protein for function analysis, the cDNA fragment *Sali*3-2c encoding the SALI3-2 fragment without the signal peptide (21–276 aa, named SALI3-2C, Figure 1) was subcloned into pET28a and transformed into *E. coli* BL21 for protein expression. The isolated fusion protein SALI3-2C with a 6×His tag was re-natured and purified on a Ni-sepharose column with a buffer containing decreasing concentrations of urea. Thrombin was then added to the purified 6×His- SALI3-2C protein to cleave off the N-terminal 6×His peptide (Figure S1). The SALI3-2C protein was then purified and maintained at 20°C for later use.

**Figure 2:**
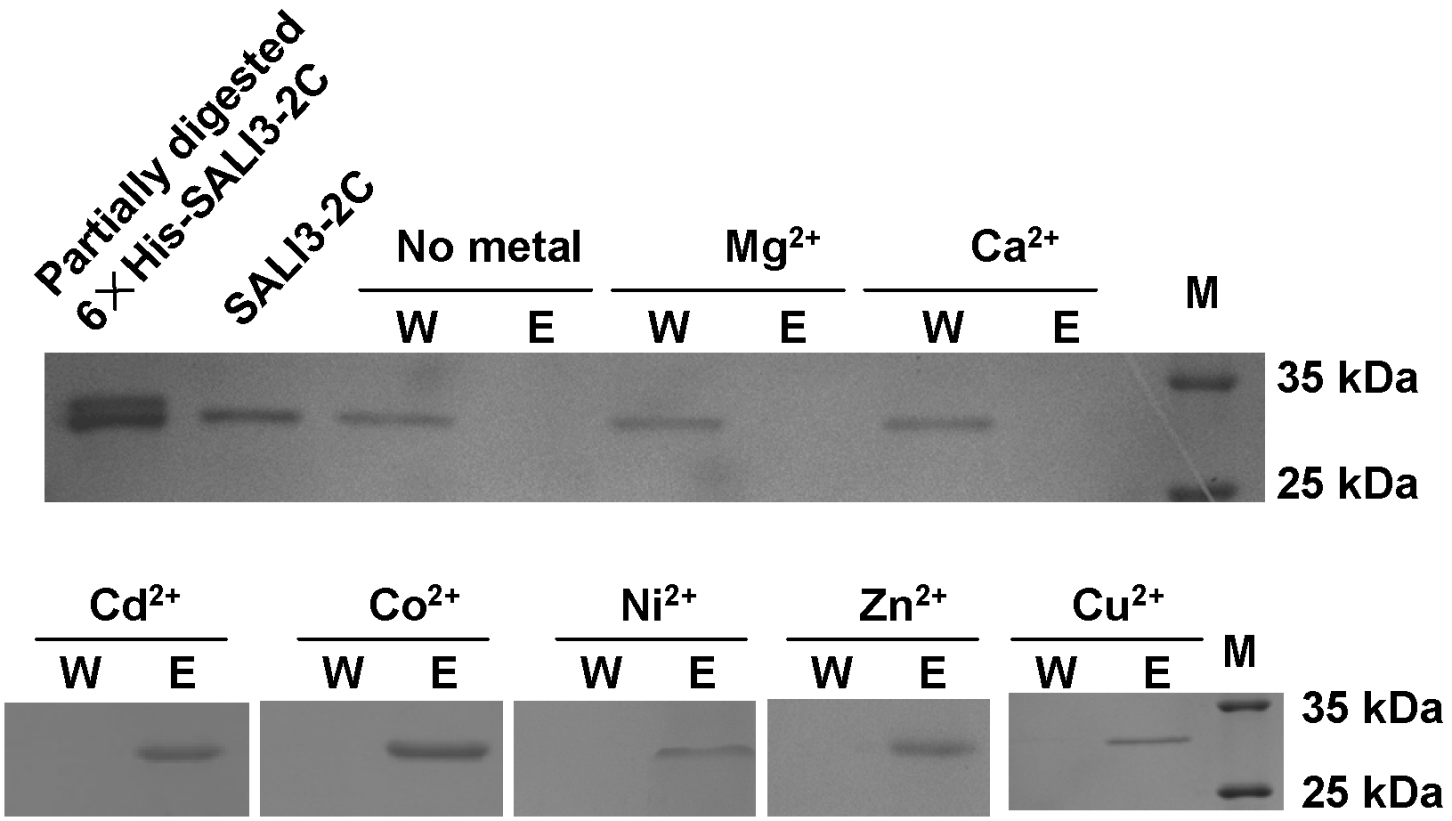
Metal-SALI3-2C binding assay using immobilized metal ion affinity chromatography (IMAC). The columns were charged with no metal, Mg^2+^, Ca^2+^, Cd^2+^, Co^2+^, Ni^2+^, Zn^2+^ or Cu^2+^. The protein was loaded onto a column that was equilibrated with 20 µM Tris–HCl buffer at pH 8.0 containing 500 mM NaCl. The unbound protein on the column was washed out with the equilibrated buffer (W). Then, the bound protein was eluted with 250 mM EDTA (E). The samples were collected, subjected to SDS-PAGE and stained with Coomassie Brilliant Blue. For the gel electrophoresis analysis, the protein marker (M), the partially digested 6×His-SALI3-2C protein and the purified SALI3-2C were also loaded.

The highly conserved Cys and Cys-His motifs in the BURP-domain in BURP family proteins indicate that these proteins potentially bind metal ions. To directly address whether SALI3-2 could chelate metal ions, the IMAC was exploited to detect whether SALI3-2C could bind metal ions under high ionic strength (500 mM NaCl). Chromatography was performed at pH 8.0 for each metal ions. The results indicate SALI3-2C was washed off in the equilibrated buffer (W) in the columns without metal ion binding (negative control) or with immobilized Ca^2+^or Mg^2+^. In the columns immobilizing Cd^2+^, Co^2+^, Ni^2+^, Zn^2+^ and Cu^2+^, SALI3-2C was retained in the column and further eluted by the elution solution (E) (Figure 2). These results indicated that SALI3-2 might bind to the soft metal ions Cd^2+^, Cu^2+^, Co^2+^, Ni^2+^and Zn^2+^ but not to the hard metal ions Ca^2+^ and Mg^2+^.

### SALI3-2-GFP is Localized in Vacuoles

**Figure 3:**
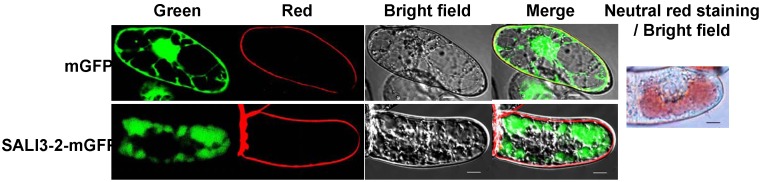
Subcellular localization of SALI3-2. BY2 cells that were expressed with SALI3-2-mGFP or mGFP were observed under confocal microscopy. Confocal fluorescence (green for mGFP, red for the membrane marked by fluorescent dyes FM4-64, and the merged images) and bright-field transmission are shown. As the control, the vacuoles are shown by neutral red staining. Scale bars = 10 µm.

To investigate the subcellular localization of SALI3-2, either the fusion gene *Sali*3-2-m*Gfp* or the m*Gfp* in pCAMBIA1302 was introduced into BY2 cells to express the SALI3-2-mGFP fusion protein or mGFP. Using the fluorescent dye FM4-64 to define the cell membrane, the subcellular localization of SALI3-2-mGFP and mGFP in transgenic BY2 cells was analyzed. The green fluorescence signal from mGFP was observed in the cytosol, nuclear matrix and/or cell membrane. And the green fluorescence of the SALI3-2-mGFP fusion protein was present in the cellular compartments that were neutral red-stained, which were mainly vacuoles (Figure 3; additional images are shown in Figure S2). These results demonstrate that SALI3-2 was targeted to cell vacuoles.

### Expression of *Sali*3-2 Enhances Cd^2+^ and Cu^2+^ Tolerance in *Arabidopsis thaliana*

The binding property of SALI3-2 with soft transition metal ions suggests that SALI3-2 might be functionally regulated by these heavy metal ions or be involved in the plant response to them. To determine whether SALI3-2 contributes to heavy metal resistance in plants, we transformed the *Sali*3-2 gene into *Arabidopsis thaliana*. Five homozygous transgenic lines were selected according to the segregation ratio of hygromycin resistance in the T2 and T3 generations and further confirmed by RT-PCR using total RNA extracted from the seedling leaves and two *Sali*3-2 -specific primers. The specific products at 830 bp were produced in the *Sali*3-2-transgenic plants but not in the plants transformed with the empty vector pCAMBIA1300 and the wild type Columbia (two lines were shown in Figure S3). No apparent differences between the phenotypes of each line grown in soil were shown (Figure S4).

**Figure 4:**
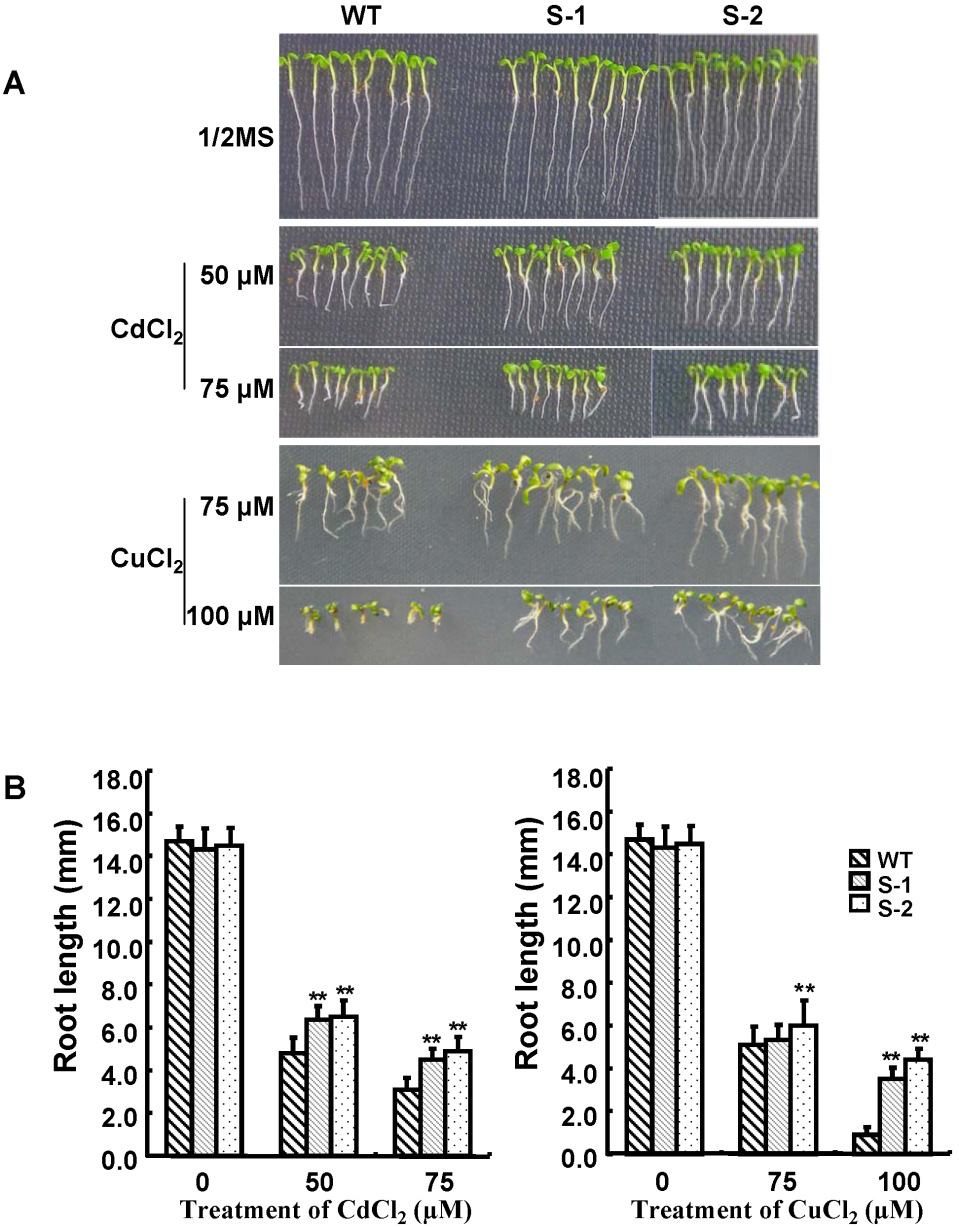
Growth of wild type and *Sali*3-2-transgenic seedlings cultured on Cd^2^^+^- or Cu^2^^+^-containing medium. The seeds were sown on 1/2 MS agar plates adding different concentrations of CdCl_2_ or CuCl_2_. Seven days after sowing, the photographs were taken (A) and the root lengths were measured (B). S-1 and S-2: two different *Sali*3-2-transgenic lines. n = 30∼40. The error bars represent SD. **P<0.01 and *P<0.05.

The T3 seeds of the homozygous transgenic lines were further used to analyze cadmium and copper tolerance. The seeds were germinated on 1/2 MS agar plates in the absence or presence of different concentrations of CdCl_2_ and CuCl_2_. The germination ratio of neither the transgenic lines nor the wild type was apparently affected by any of the treatments (Figure S5), but a significant reduction of growth of both the wild-type and transgenic seedlings was caused in the presence of CdCl_2_ or CuCl_2_ at the tested concentration. However, compared to the wild type, the transgenic plants displayed less root growth inhibition (Figure 4). Therefore, the *Sali*3-2-expressing seedlings were more tolerant to cadmium and copper exposure.

### *Sali*3-2-transgenic *Arabidopsis thaliana* Plants Delivers Less Cadmium and Copper to the Shoot after Corresponding Heavy Metal Ions Exposure

**Figure 5:**
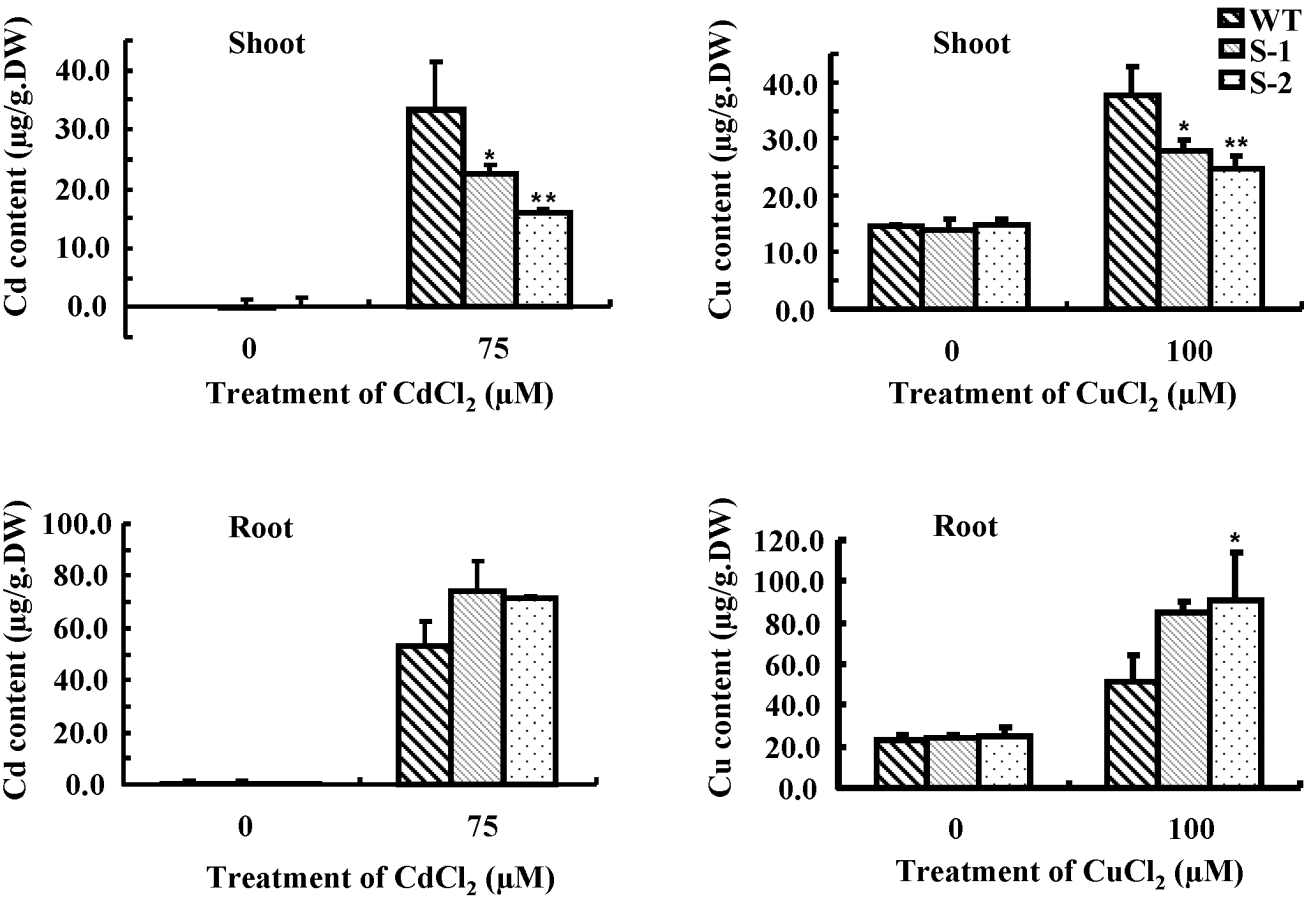
Contents of Cd and Cu in the shoots and the roots of *Sali*3-2-transgenic plants. Wild-type and *Sali*3-2-transgenic seedlings were grown for 2 weeks on 1/2 MS agar plates and were then transferred into 1/2 MS solution medium containing 75 µM CdCl_2_ or 100 µM CuCl_2_ for an additional 2 d of growth. The shoots and the roots were harvested to measure the Cd or Cu contents using ICP-OES. A representative from 3 independent experiments is shown. The error bars represent SD. **P<0.01 and *P<0.05.

We measured the Cd and Cu contents of the wild type and *Sali*3-2-transgenic plants. Compared to the wild type, the transgenic plants contained less Cd or Cu in the shoots, and more in the roots after treatment with CdCl_2_ or CuCl_2_ (Figure 5). The results indicate that the transgenic plants deliver less Cd or Cu to the shoot than the wild type plants do when they experienced Cd^2+^ or Cu^2+^ stresses.

### *Sali*3-2 is Expressed at Higher Level in the Roots than in the Upper Ground Tissues in the Native Soybean

**Figure 6:**
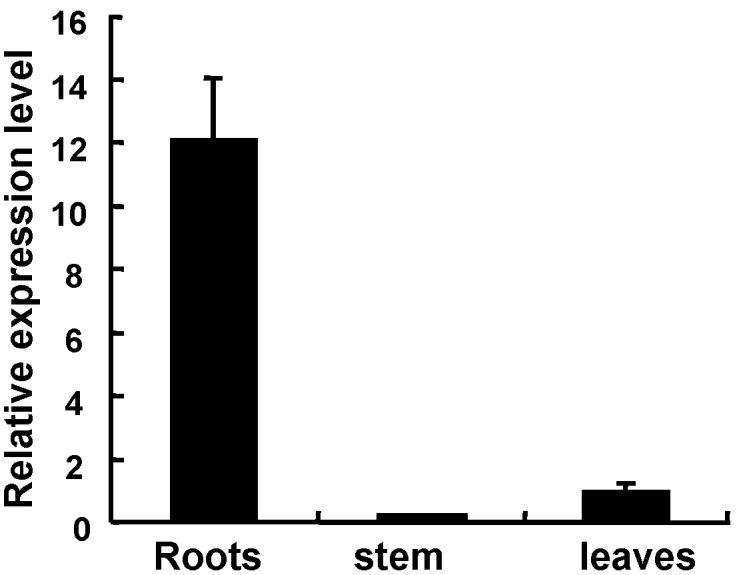
Expression level of *Sali*3-2 in different tissues of soybean seedlings. The expression level in the leaves was set to 1, and the *Tubulin* gene was used as the internal control. The error bars represent SD.

Quantitative RT-PCR (qRT-PCR) assays showed that *Sali*3-2 was expressed in the roots at a high level and in the stems and leaves at a low level (Figure 6), indicating that *Sali*3-2 may play a special role in the root in native soybean.

## Discussion

In recent years, BURP proteins have attracted attention due to their diverse functions in plant development and stress responses. Here, we cloned the BURP gene *Sali*3-2 from soybean and studied the biochemical functions and mechanism of the SALI3-2 protein in heavy metal resistance. The results that we obtained suggest a role for SALI3-2 in heavy metal conjugation and resistance. First, SALI3-2 can interact with heavy metal ions (Cu^2+^, Cd^2+^, *etc.*) *in vitro*. Second, *Sali*3-2-transgenic *Arabidopsis thaliana* seedlings were more tolerant to Cu^2+^ or Cd^2+^ exposure than the wild type were.

Eukaryotic organisms have developed multiple mechanisms for tolerance to heavy metal ion stress during their evolution. One mechanism is to reduce the amount of ions that are transported into cells through the cell wall or by cell surface binding. For example, yeast could prevent copper-induced toxicity by precipitating copper ions on the cell membrane by forming copper sulfide (CuS) mineral lattices [26]. Another mechanism is to maintain ion homeostasis. Ion transporters, such as the copper-transporting P-type ATPases [27], [28], [29] and the ABC transporter [30], can transport metal ions into certain compartments. Third, intracellular heavy metal ions can be detoxified by organic acids or heavy metal-binding peptides [31]. Metallothioneins (MTs) and phytochelatins (PCs) are two major types of heavy metal-binding peptides [32], [33], [34], [35], [36]. MTs have repeated sequence motifs of either Cys-x-Cys or Cys-xx-Cys [37], and PCs have the general structure of (γ-Glu- Cys)n-Gly (n = 2∼11) [38], [39]. These Cys-rich proteins potentially interact with metals such as Cd^2+^, Cu^2+^, or Zn^2+^ of high affinity and with several binding sites *via* their cysteine residues [19]. These proteins provide a transient storage form for the ions and they may play a primary role in heavy metal ion sequestration in vacuoles by binding metal ions [40], while the tonoplast transporters ABCCs of the ABC transporter family most likely play an important assisting role in the vacuolar transportation of the peptide-binding heavy metal ions or chelator-ions complex [30].

In general, regarding to the binding of metal ions to proteins, soft metal ions can form strong bonds with soft Lewis bases such as the thiols of cysteine residues and the imidazolium nitrogens of histidine residues. In contrast, hard metal ions bind strongly to the hard Lewis bases, such as the carboxyl oxygens of glutamate or aspartate residues [41]. SALI3-2 is a BURP family member, in its C-termini possessing a conserved BURP domain with four conserved Cys-His repeats and two other Cys residues [1], [3]. We previously studied the interaction of Cu^2+^ with SALI3-2 and found that Cu^2+^ binds to SALI3-2 with a relatively high affinity and alters the conformation of SALI3-2 [20]. In this study, we further confirm that SALI3-2 not only interacts with Cu^2+^ but also interacts broadly with other soft metal ions, such as Cd^2+^, Co^2+^, Zn^2+^ and Ni^2+^. Furthermore, SALI3-2 is localized in vacuoles. Therefore, SALI3-2 might function similar to MTs and PCs, playing roles in sequestrating heavy metal ions in vacuoles by binding the ions *via* Cys and His residues and alleviating the toxicity to cells due to these ions, possibly explaining the roots of *Sali*3-2-transgenic seedlings displaying less inhibition than that of the wild type under cadmium and copper stress. Consecutively, in the *Sali*3-2-transgenic plants the enhanced retention of metals in root vacuoles may lead plants to accumulate more metal ions in root and reduce the amount of Cd^2+^ or Cu^2+^ that is delivered to shoot [42].

However, the number of binding sites of SALI3-2 with Cu^2+^ is only 1.6 [20]. With such a low bound amount, the mechanism of SALI3-2 in heavy metal ion tolerance would not be similar to that of MTs or PCs in providing a transient storage form for the ions. Another possibility is that SALI3-2 promotes the transport of Cu^2+^, Cd^2+^, *etc*. into vacuoles by regulating the action of ion transporters in the vacuole membrane (*i.e.*, the ABCC type proteins [30]) and by maintaining ion homeostasis. To verify this hypothesis, further studies should be performed, for example, identifying the interaction partners of SALI3-2, and analyzing the accumulated amount of heavy metal in vacuoles.

Our qRT-PCR assays showed that *Sali*3-2 was expressed at high levels in roots but at low levels in the upper ground tissues in native soybean. This result is consistent with that reported by Granger *et al*. based on expressed sequence tags (ESTs) analyses [2]. In that paper, the number of ESTs of *Sali*3-2 is also small in pod. These findings also suggest a function of SALI3-2 in sequestering heavy metal ions in the native soybean root. Our results support the exploration of the use of *Sali*3-2 for the bioaccumulation of metal ions in the roots to protect aerial parts from excess metal contamination.

In summary, we found that SALI3-2 is localized in vacuoles and can bind heavy metals (Cd^2+^, Cu^2+^, ***etc.***) *in vitro*. The heterologous expression of *Sali*3-2 enables plants to sequester Cd^2+^ or Cu^2+^ in the roots and reduce the amount of heavy metals that are transported to the shoot, further enhance the tolerance to excessive copper and cadmium stresses. Therefore, our findings suggest that SALI3-2 may play a role in heavy metal conjugation and resistance in this overexpression system. A possible function of SALI3-2 that is different from those of other BURP proteins is presented.

## Supporting Information

Figure S1 **SDS-PAGE of protein extracts.** M: marker; 1. Purified SALI3-2C; 2 and 3. Purified 6×His-SALI3-2C; 4. 6×His-SALI3-2C partially digested by thrombin for 2 h.
(TIF)

Figure S2 **Subcellular localization of SALI3**-**2.** BY2 cells that were expressed with SALI3-2-mGFP or mGFP were observed under confocal microscopy. Confocal green fluorescence and bright-field transmission are shown. Scale bars = 10 µm.
(TIFF)

Figure S3 **Expression of** ***Sali*** **3**-**2 in transgenic plants as detected by RT-PCR.** Total RNA was extracted from the young leaves of 3-week-old plants. RT-PCR was performed using *Tubulin* as the control. WT: wild-type plants; 00: plants that were transformed with the empty vector pCAMBIA1300; S-1 and S-2: two different *Sali*3-2-transgenic lines.
(TIFF)

Figure S4 **Phenotype of transgenic plants grown in soil.** DAS: days after sown.
(TIF)

Figure S5 **Seeds germination ratio of different lines on Cd^2^** ^+^ **- or Cu^2^** ^+^ **-containing medium.** More than 60 seeds of each line were tested in each independent treatment. A representative from 3 independent experiments is shown. The error bars represent SD.
(TIF)
