# Correction: Expression of a Vacuole-Localized BURP-Domain Protein from Soybean (SALI3-2) Enhances Tolerance to Cadmium and Copper Stresses

**DOI:** 10.1371/journal.pone.0107337

**Published:** 2014-08-29

**Authors:** The PLOS ONE Staff

The authors are listed out of order. Please view the correct author order, affiliations, and citation here:

Yulin Tang^1^, Yan Cao^2^, Zhan Gao^1^, Zhonghua Ou^1^, Yajing Wang^1^, Jianbin Qiu^2^, Yizhi Zheng^1^

1 Shenzhen Key Laboratory of Microbial and Gene Engineering, College of Life Sciences, Shenzhen University, Shenzhen, Guangdong, People’s Republic of China

2 The Key Laboratory for Marine Bioresource and Eco-environmental Science, College of Life Sciences, Shenzhen University, Shenzhen, Guangdong, People’s Republic of China

Tang Y, Cao Y, Gao Z, Ou Z, Wang Y, et al. (2014) Expression of a Vacuole-Localized BURP-Domain Protein from Soybean (SALI3-2) Enhances Tolerance to Cadmium and Copper Stresses. PLoS ONE 9(6): e98830. doi:10.1371/journal.pone.0098830
