# Supplementary figures and images for: Expression of a Vacuole-Localized BURP-Domain Protein from Soybean (SALI3-2) Enhances Tolerance to Cadmium and Copper Stresses
Source: PLoS One. 2014 Jun 5;9(6):e98830. doi: 10.1371/journal.pone.0098830 (PMC4047006; doi:10.1371/journal.pone.0098830)

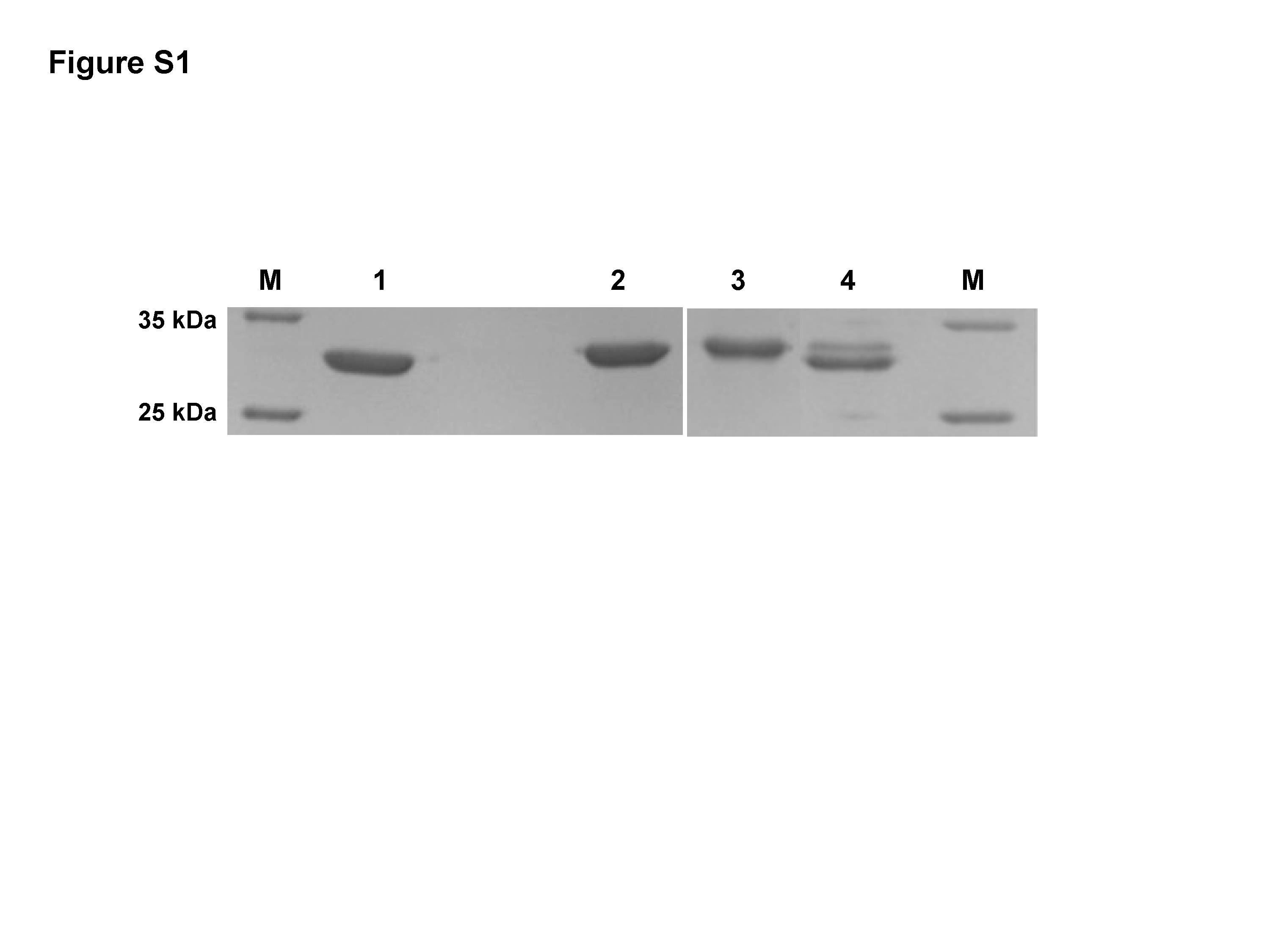

Supplement: Figure S1 — SDS-PAGE of protein extracts. M: marker; 1. Purified SALI3-2C; 2 and 3. Purified 6×His-SALI3-2C; 4. 6×His-SALI3-2C partially digested by thrombin for 2 h. (TIF) [file pone.0098830.s001.tif]

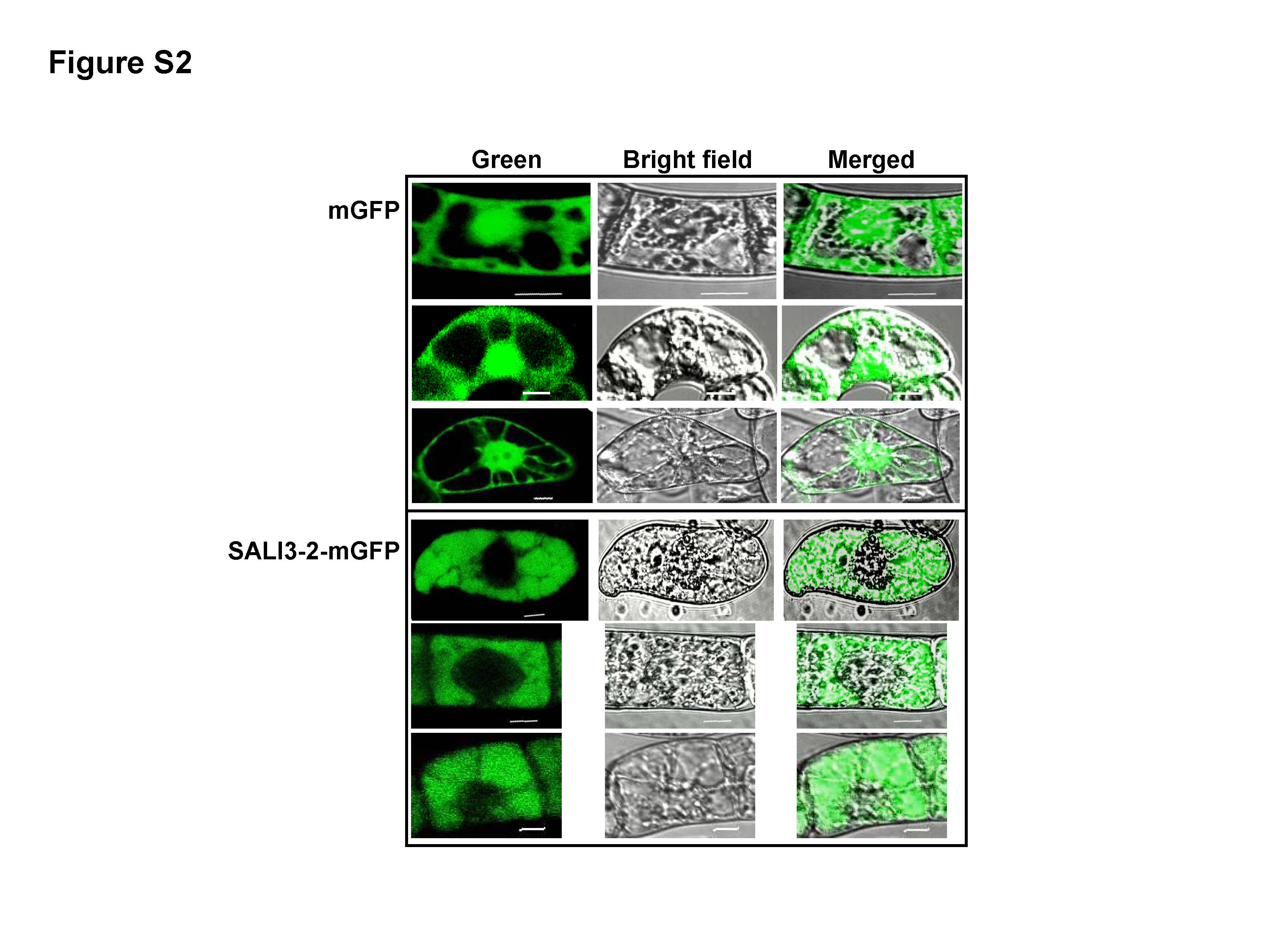

Supplement: Figure S2 — Subcellular localization of SALI3-2. BY2 cells that were expressed with SALI3-2-mGFP or mGFP were observed under confocal microscopy. Confocal green fluorescence and bright-field transmission are shown. Scale bars = 10 µm. (TIFF) [file pone.0098830.s002.tif]

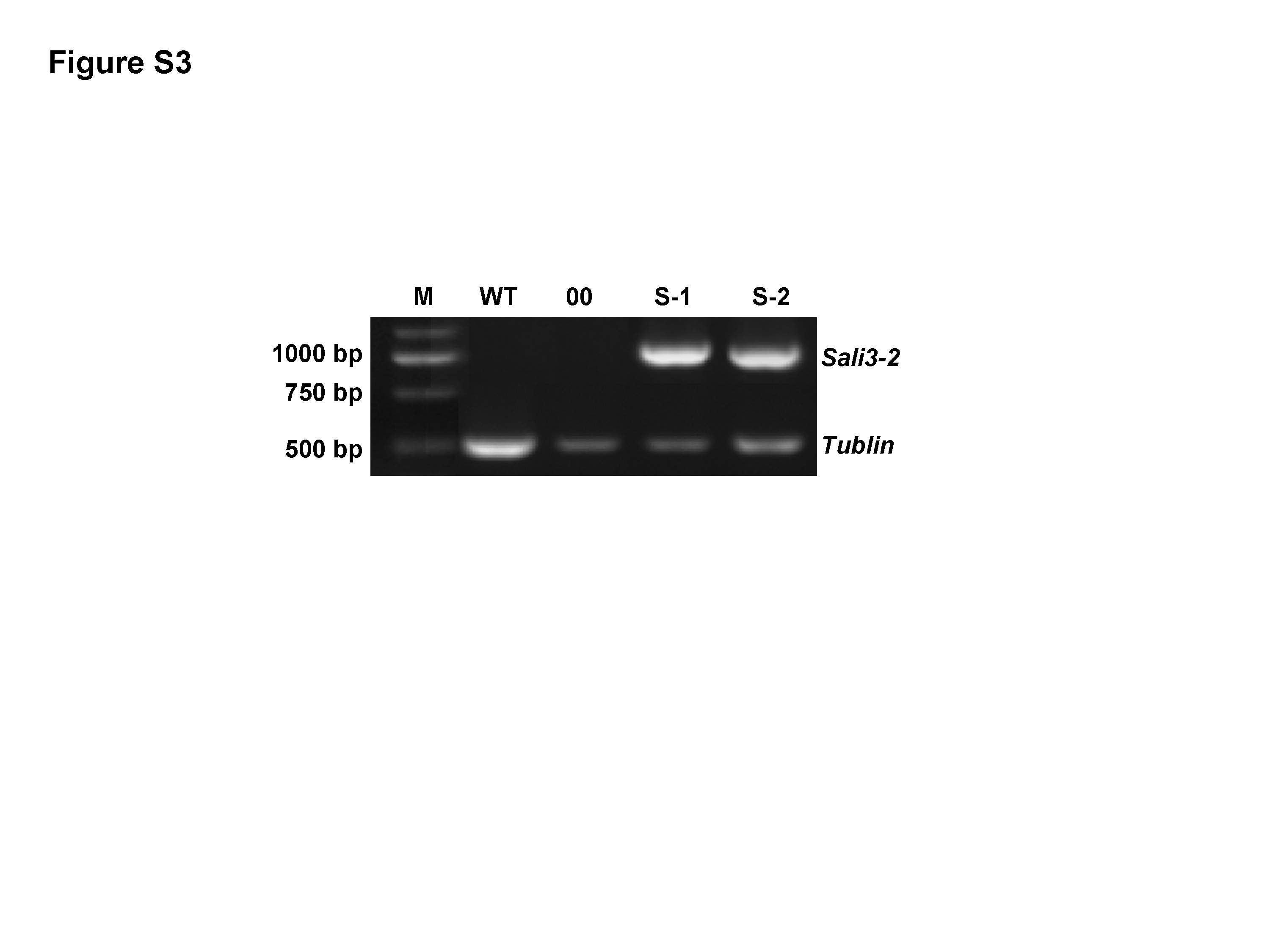

Supplement: Figure S3 — Expression of Sali 3-2 in transgenic plants as detected by RT-PCR. Total RNA was extracted from the young leaves of 3-week-old plants. RT-PCR was performed using Tubulin as the control. WT: wild-type plants; 00: plants that were transformed with the empty vector pCAMBIA1300; S-1 and S-2: two different Sali3-2-transgenic lines. (TIFF) [file pone.0098830.s003.tif]

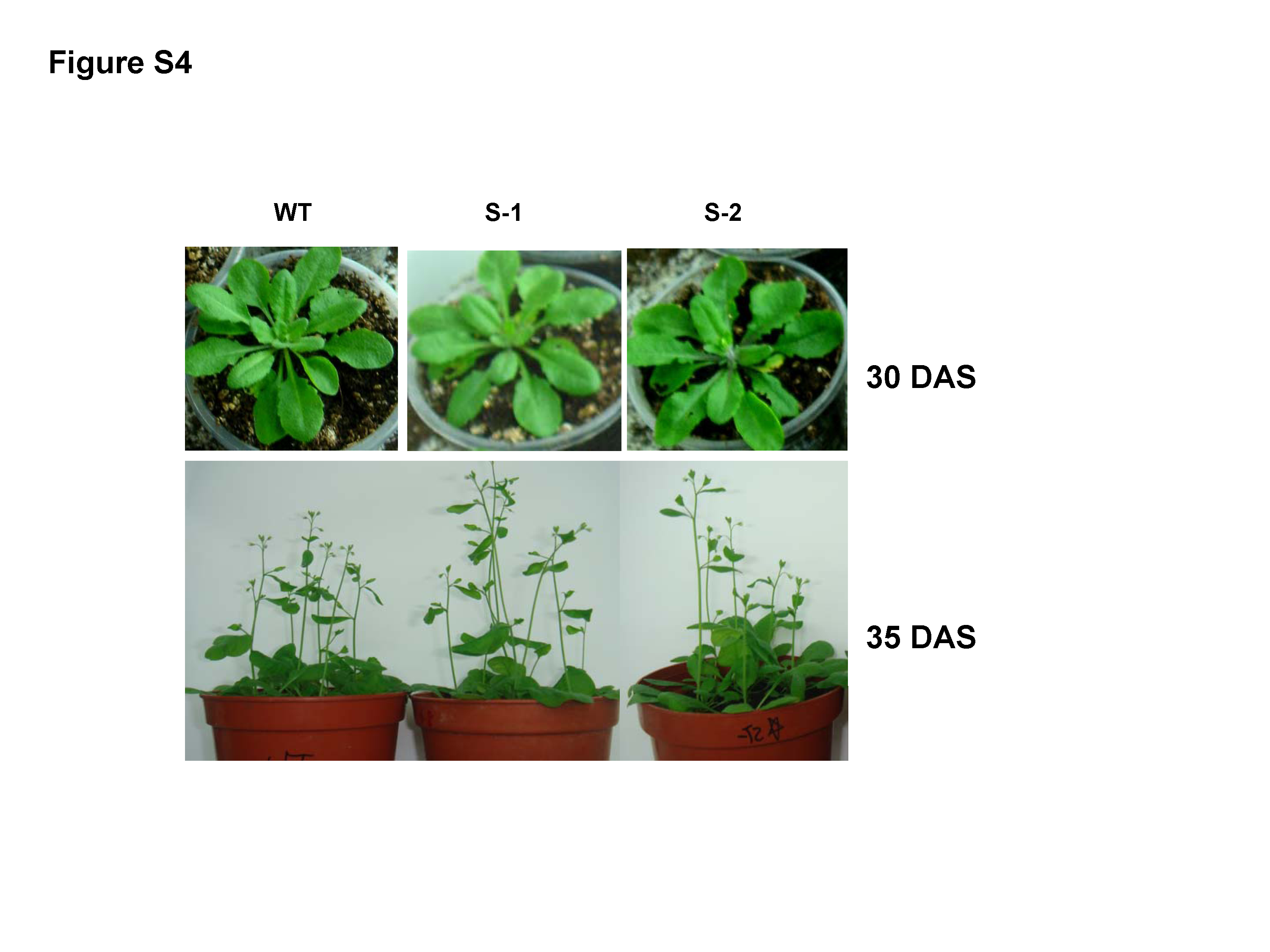

Supplement: Figure S4 — Phenotype of transgenic plants grown in soil. DAS: days after sown. (TIF) [file pone.0098830.s004.tif]

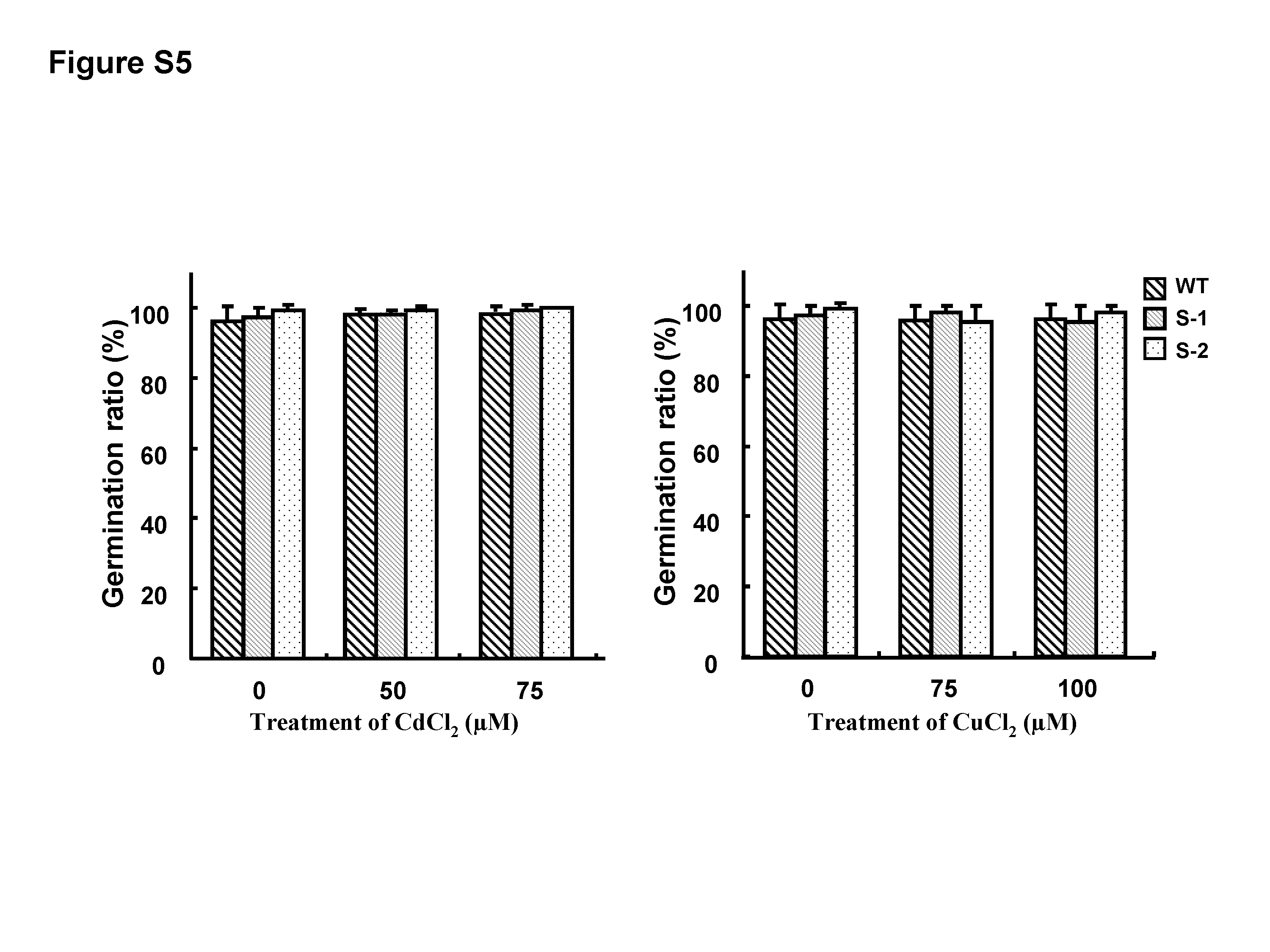

Supplement: Figure S5 — Seeds germination ratio of different lines on Cd2 + - or Cu2 + -containing medium. More than 60 seeds of each line were tested in each independent treatment. A representative from 3 independent experiments is shown. The error bars represent SD. (TIF) [file pone.0098830.s005.tif]
